# mHealth Apps Using Behavior Change Techniques to Self-report Data: Systematic Review

**DOI:** 10.2196/33247

**Published:** 2022-09-09

**Authors:** Maria Aguiar, Maria Trujillo, Deisy Chaves, Roberto Álvarez, Gorka Epelde

**Affiliations:** 1 Vicomtech Foundation Basque Research and Technology Alliance Donostia-San Sebastián Spain; 2 Multimedia and Computer Vision Group Universidad del Valle Cali Colombia; 3 Department of Electrical Systems and Automation Universidad de León León Spain; 4 Biodonostia Health Research Institute eHealth Group Donostia-San Sebastián Spain

**Keywords:** mobile health, mHealth, behavior change techniques, adherence, app, mobile health interventions, behavior

## Abstract

**Background:**

The popularization of mobile health (mHealth) apps for public health or medical care purposes has transformed human life substantially, improving lifestyle behaviors and chronic condition management.

**Objective:**

This review aimed to identify behavior change techniques (BCTs) commonly used in mHealth, assess their effectiveness based on the evidence reported in interventions and reviews to highlight the most appropriate techniques to design an optimal strategy to improve adherence to data reporting, and provide recommendations for future interventions and research.

**Methods:**

We performed a systematic review of studies published between 2010 and 2021 in relevant scientific databases to identify and analyze mHealth interventions using BCTs that evaluated their effectiveness in terms of user adherence. Search terms included a mix of general (eg, data, information, and adherence), computer science (eg, mHealth and BCTs), and medicine (eg, personalized medicine) terms.

**Results:**

This systematic review included 24 studies and revealed that the most frequently used BCTs in the studies were feedback and monitoring (n=20), goals and planning (n=14), associations (n=14), shaping knowledge (n=12), and personalization (n=7). However, we found mixed effectiveness of the techniques in mHealth outcomes, having more effective than ineffective outcomes in the evaluation of apps implementing techniques from the feedback and monitoring, goals and planning, associations, and personalization categories, but we could not infer causality with the results and suggest that there is still a need to improve the use of these and many common BCTs for better outcomes.

**Conclusions:**

Personalization, associations, and goals and planning techniques were the most used BCTs in effective trials regarding adherence to mHealth apps. However, they are not necessarily the most effective since there are studies that use these techniques and do not report significant results in the proposed objectives; there is a notable overlap of BCTs within implemented app components, suggesting a need to better understand best practices for applying (a combination of) such techniques and to obtain details on the specific BCTs used in mHealth interventions. Future research should focus on studies with longer follow-up periods to determine the effectiveness of mHealth interventions on behavior change to overcome the limited evidence in the current literature, which has mostly small-sized and single-arm experiments with a short follow-up period.

## Introduction

### Overview

In modern society, the rushed lifestyle and excessive adulteration in food products have caused health-related disorders, making them an inevitable part of modern life [1]. This is associated with the development of noncommunicable diseases, which are related to 16 million premature deaths per year worldwide. The treatment of these lifestyle-related disorders demands long-term clinical help and can last a lifetime [2].

Besides, smartphones have become an essential tool in our daily lives, impacting 7.2 billion users worldwide with more than 70% of them in low- and middle-income countries [3]. Smartphone sensor technology has significantly improved and become more stable for collecting real-time data, which can be saved and processed for multiple analyses, making it possible to monitor our health through mobile health (mHealth) apps [4,5].

Recently, the popularization of mHealth apps for public health or medical care purposes have transformed human life substantially. Strategies such as reminders, counselling, reinforcement, or education have been used to improve people’s adherence to the app, thus improving lifestyle behaviors [6] and chronic condition management (CCM). These strategies are known as behavior change techniques (BCTs).

There is a need to improve the adherence to one’s well-being, regular health monitoring, and expert involvement [7]. The World Health Organization (WHO) estimated that in high-income countries the average adherence rate is 50% in patients with chronic medical illness [8], with even lower rates in low-income countries. It considers the extent to which a person’s behavior—taking medication, following a diet, or making lifestyle changes—corresponds to recommendations agreed upon with a health professional directly or through a mobile app. Nonadherence leads to considerable morbidity, mortality, and avoidable health care costs [9], and it may be caused by people’s intentional or unintentional behaviors. Intentional nonadherence refers to deciding not to report data based on the person’s perceptions such as incomplete disease-related knowledge. In contrast, unintentional nonadherence means that the person intends to report data but fails because of forgetfulness or carelessness. Awareness and proper screening of these intentional and unintentional determinants for the target population are necessary to design and develop tailored solutions to ensure a methodology that improves adherence to data reporting.

mHealth has the potential to improve lifestyle and CCM, and can be rapidly adopted on a large scale and at low cost [10], but inconsistent findings have been reported on its effectiveness. Table 1 summarizes the systematic reviews conducted in the literature on the effectiveness of mHealth interventions with BCTs in distinct contexts and populations.

Although the reviews include mHealth studies for a specific population, activity, or disease, most of these studies evaluated the effectiveness in terms of the results obtained for the intervention’s objective. Because of this, it is difficult to discern whether the intervention was ineffective due to a lack of adherence by participants or the combination of 35 BCTs being inadequate for the problem addressed. Therefore, the motivation of this systematic review is to identify current studies that have specifically reported their results in terms of adherence to extract the most used BCTs among the effective studies. This will help to design an adherence-focused strategy combining these BCTs.

**Table 1:** Systematic reviews were examined, describing the number of studies included and their research objective.

Authors	Studies, n	Objective
Schorr et al [11]	26	Identify studies using mHealth^a^ for secondary CVD^b^ prevention that focus on lifestyle behavior change and medication adherence
Akinosun et al [12]	25	Identify and measure the effectiveness of digital technology interventions (eg, mobile phones, the internet, software applications, or wearables) in randomized controlled trials and determine which behavior change constructs are effective at achieving risk factor modification in patients with CVD
Godinho et al [13]	29	Examine the implementation and evaluation of mHealth to support the integration of people-centered health services in the World Health Organization Western Pacific Region
Monteiro-Guerra et al [14]	17	Study real-time PA^c^ coaching mobile apps with personalization mechanisms
Wang et al [15]	17	Evaluate the effectiveness of mHealth interventions for the treatment and management of diabetes and obesity reported in reviews and meta-analyses to provide recommendations for future interventions and research
Thomas Craig et al [16]	30	Identify context-aware digital behavior change interventions that provide individualized interventions to improve health
Bearne et al [17]	4	Identify apps that facilitate PA for adults with rheumatoid arthritis and compare the quality and content of these apps to incorporate relevant BCTs^d^ against recommendations for cardiorespiratory, resistance, flexibility, neuromotor PA, and exercise
Kalke et al [18]	30	Identify breast cancer apps that support behavior change and assess the extent to which these apps address cancer care content
Tighe et al [19]	7	Identify digital platformlike interventions and examine their potential for supporting self-management of noncommunicable diseases and health behavior change
Armitage et al [20]	9	Estimate the efficacy of app-based interventions designed to support medication adherence and investigate which BCTs used by these apps are associated with efficacy
Pfaeffli Dale et al [21]	7	Determine the effectiveness of mHealth interventions on behavioral lifestyle changes and medication adherence for CVD self-management

^a^mHealth: mobile health.

^b^CVD: cardiovascular disease.

^c^PA: physical activity.

^d^BCT: behavior change technique.

### Background

**Figure 1:**
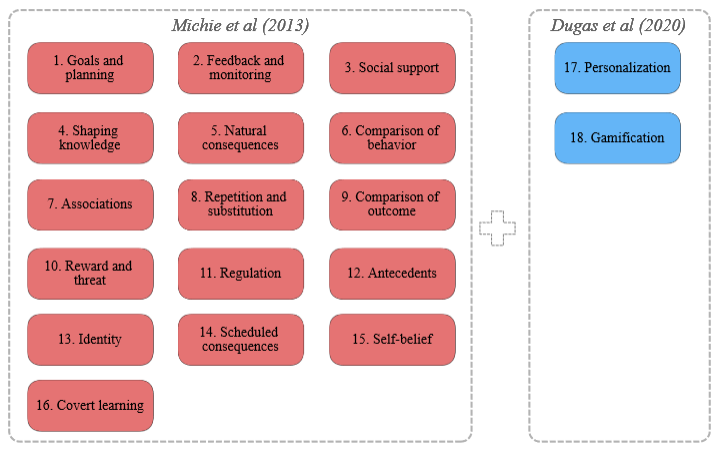
Taxonomy: behavior change techniques categories.

*mHealth* is a term used for mobile apps and other wearable devices that collect and monitor medical information from users [22]. Data collected from their routine leads to the accumulation of data depending on the number of users and how often they report data manually (eg, questionnaires) and through their wearable sensors. Therefore, the use of big data analytics on mHealth may be promising to provide medical information, improve people’s well-being in nonclinical and clinical settings [23], and improve access to quality care and timely monitoring at an affordable cost with enriched outcomes. Within mHealth apps, BCT refers to an observable, replicable, and irreducible component of an intervention designed to alter or redirect causal processes that regulate behavior (eg, feedback, self-monitoring, and reinforcement) [24]. BCTs are coded using an established taxonomy of 93 techniques provided in “A Taxonomy of Behaviour Change Techniques Used in Interventions” [25]—for which a standardization has been proposed—and were initially grouped into 16 categories [26,27]. Besides, Dugas et al [27] extended the taxonomy of BCTs in 2020 with 2 additional categories, personalization and gamification, comprising 9 BCTs. Figure 1 shows the final taxonomy with 18 categories that will be used in this study.

*Adherence* is defined by the WHO [8] as “the extent to which a person’s behaviour – taking medication, following a diet, and/or executing lifestyle changes – corresponds with agreed recommendations from a health care provider.” Moreover, from a technical point of view, adherence is defined as the developer’s expectations, referring to the degree to which the user’s activity within the app matches the pattern of activity that was intended by the developers, differing from the definition of usage that refers to the level of activity within an app. For example, a user who completes 5 modules in a program will obtain 100% usage on the modules’ metric of usage. However, if these modules were supposed to be completed weekly and the user only completed 3 of these on time, the user achieved 60% on the modules’ adherence metric. On the other hand, if a user completes all the activities in an app by the time they are scheduled, the user adherence is 100% [7]. If people do not report data as often as expected or stop using the app, the quantity and homogeneity of data to be processed will be reduced, producing lower quality outcomes. We will consider both definitions when reviewing the efficacy of the studies.

## Methods

### Research Questions

The objective of our systematic review is to identify and analyze relevant studies on BCTs used in mHealth interventions, focusing on the effectiveness of the BCTs on use and paying special attention to improve adherence to data reporting. Hence, the research questions (RQs) that have guided this review are:

- RQ1: What BCTs are most commonly used in the context of mHealth apps? How can these techniques be classified?
- RQ2: What is the performance of these techniques concerning end-user adherence?
- RQ3: Are BCTs (personalization, feedback, and monitoring strategies) useful for improving adherence to data reporting in mHealth apps?

### Search Strategy

This systematic review was conducted according to the PRISMA (Preferred Reporting Items for Systematic Reviews and Meta-Analyses) statement [28]. Details of the search strategy are given below.

#### Eligibility Criteria

Eligible studies were peer-reviewed articles published in English from January 1, 2010, to December 2021. With the growth of technology used for mHealth, the selected time allowed us to assess studies using the most relevant technologies. Participants in the studies were patients of any age, both healthy and with any type of chronic disease. Moreover, studies with interventions using health practices supported by any type of mobile device were eligible for inclusion. Besides, interventions could include multiple delivery methods and nondigital elements. The result assessed was adherence focusing on any outcome (eg, physical activity, medication adherence, and data reporting) measured by any metric during any follow-up period.

#### Selection of Sources

The scientific databases selected for the review were Scopus, PubMed, Web of Science, and IEEE Xplore. The combination of these databases provides comprehensive coverage of publications in the context of medical informatics, high relevance, and a complete advanced search. Search strings and search methods were consistent across all databases.

#### Search Terms

A preliminary literature review resulted in the first search equation, which aimed to find the different applications of mHealth in personalized medicine. This allowed us to identify relevant keywords and search terms to refine the search equation in each iteration by focusing on relevant topics such as adherence and data reporting but without limiting it to the field of personalized medicine. Finally, behavior change was found to be a popular aspect of improving adherence and hence the effectiveness of studies. We only included the term “behaviour change” because it is not limited to techniques, as there are different theories and models that contain them. As a result, four search iterations were performed. Those iterations are shown in Table 2.

**Table 2:** Search equations, data source, and total records per query.

Search equation and source			Total records, n
**1 (“mHealth” AND “personalised medicine”)**			
	Scopus	124	
	PubMed	19	
	Web of Science	4	
	IEEE Xplore	15	
**2 (“mobile health” OR “mHealth”) AND “adherence” AND “personalised medicine”)**			
	Scopus	23	
	PubMed	2	
	Web of Science	0	
	IEEE Xplore	5	
**3 (“mHealth” AND (“data” OR “information”) AND “registration” AND “adherence”)**			
	Scopus	52	
	PubMed	152	
	Web of Science	35	
	IEEE Xplore	1	
**4 (“mobile health” OR “mHealth”) AND “adherence” AND “behaviour change”)**			
	Scopus	156	
	PubMed	120	
	Web of Science	23	
	IEEE Xplore	9	

### Inclusion/Exclusion Criteria

After the initial gathering and screening of studies, articles were selected based on predefined eligibility criteria.

#### Inclusion Criteria

The included studies meet the criteria of being an mHealth intervention. In addition, they fulfill at least one of the following conditions:

- The study implements at least one BCT.
- The study compares several BCTs.
- The study evaluates the BCTs using at least one adherence metric.

#### Exclusion Criteria

The excluded studies meet at least one of the following conditions that aim to discard irrelevant studies for this systematic review:

- The study does not address the use of BCTs in the context of mHealth or vice versa.
- The study does not present at least one of its results in terms of adherence.
- The study was not published in between the years 2010-2021.
- The study does not belong to one of these categories: journal paper, conference paper, or review.
- The study was not peer reviewed.
- The study is not written in English or Spanish.

### Data Extraction and Coding of BCTs

PRISMA [28] guidelines were used for data extraction. We gathered information about the study background (year, authors, etc), eligibility criteria (population), the number of participants, intervention description, technology, and results.

The behavioral strategy used in each study was identified and coded using the taxonomy of 18 categories introduced in the Background section to answer RQ1. To perform correct identification and coding, free taxonomy training was received using materials available online [29]. Results using any adherence assessment metric were extracted to solve RQ2. Finally, to answer RQ3, a more in-depth analysis was performed in the Discussion section about what was found to answer the previous RQs.

## Results

This section presents the results obtained from the methodology described in the Methods. First, an overview of the selected studies and their main characteristics is presented, and then an analysis of these studies is performed.

### Search Results and Study Selection

**Figure 2:**
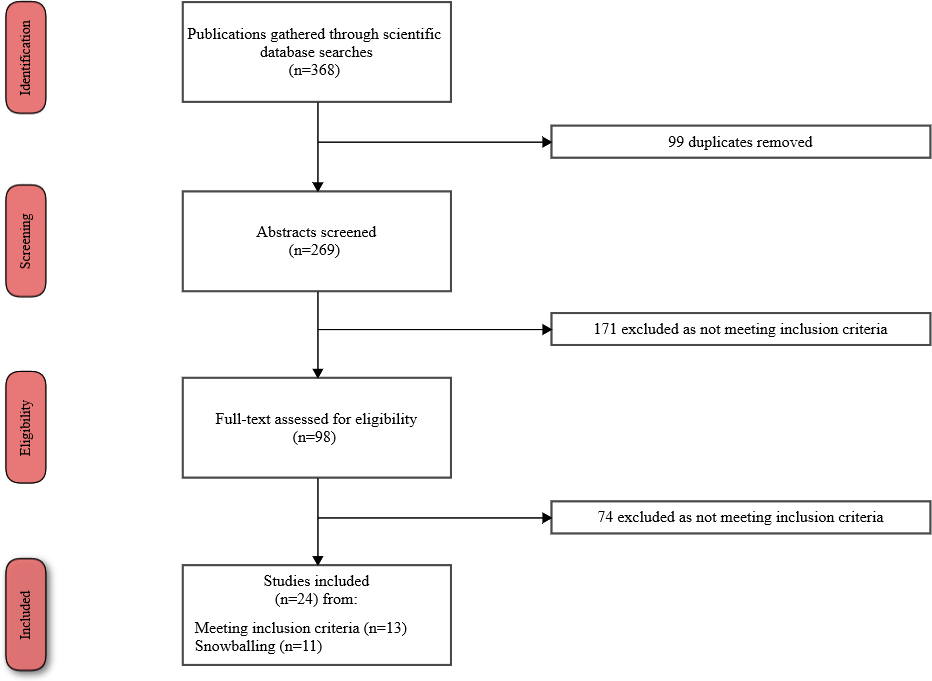
PRISMA (Preferred Reporting Items for Systematic Reviews and Meta-Analyses) flowchart.

The search results and study selection are summarized in the PRISMA flowchart in Figure 2. A total of 368 studies were obtained from the search engines. Subsequently, 88 duplicate studies obtained from the combination of the databases’ results were excluded using the Mendeley tool. In the screening step, we had 269 studies, of which 171 were excluded for not meeting the inclusion criteria after abstract review. In the eligibility step, we had 98 studies, of which 74 were excluded for not meeting the inclusion criteria after full-text review. Finally, 13 studies were included; however, the snowball strategy was applied to the systematic reviews listed in Table 1, resulting in 11 additional studies. These studies were not found in our initial search because they did not use the term adherence in their title, abstract, or keywords.

### Outcome and Characteristics

We summarized the selected 24 studies [30-53] to understand which technologies and BCTs were most frequently used in mHealth research. Data extracted from each study following the methodology defined in the Data Extraction and Coding of BCTs section are presented in Table S1 in Multimedia Appendix 1 [30-53]. The first column refers to the study identifier, the next two columns specify the sample, and the remaining four columns correspond to the duration, description, technology, and evaluation of the intervention.

We observed in the reviewed papers that the most targeted behavior was medication adherence—present in 67% (n=16) of the studies—which is important in preventing rehospitalization, morbidity, mortality, and increased health care costs [54]. We also found that the most frequent population was patients diagnosed with chronic conditions, participating in 83% (n=20) of the studies, and the most popular form of technology in the studies was the use of apps, present in 71% (n=17) of the studies, with wearable support becoming popular. Still, 29% (n=7) of the studies examined the effects of SMS text message–based interventions, indicating that simpler health interventions delivered by text remain popular. With respect to the techniques, the most frequently used BCTs in the studies were feedback and monitoring (n=20, 83%), goals and planning (n=14, 58%), associations (n=14, 58%), and shaping knowledge (n=12, 50%). On the other hand, personalization (n=7, 29%) was approached in a simple way, considering their capabilities, including tailoring to demographic information, health status (eg, alcoholic or nonalcoholic), and time of notifications. On average, 4 BCTs are included in each mHealth intervention; a minimum of 1 and maximum of 8 techniques were used per study. This is shown in Table S2 in Multimedia Appendix 2 [30-53] where the BCTs used by each study are presented.

Regarding adherence evaluation metrics, it is worth mentioning that different methods are used for this measurement, from daily use of the mobile app to the Morisky Medication Adherence Scale. To analyze these studies homogeneously, a cutoff point for effectiveness in terms of adherence rate was considered. High adherence was defined by an adherence rate ≥80% and nonadherent as an adherence rate <80%. This cut point is conventional in the adherence literature [55,56] and is considered crucial for the effectiveness of long-term therapy [57]; however, it is interesting to note that many studies used the arbitrary threshold of 80% [58], indicating that the optimal cut point for adherence ranged from 58% to 85%.

Moreover, after the BCTs used in each study were identified, we classified the studies into effective and ineffective using the cut point of 80% to gain further insight about why some interventions yielded significant improvements. As a result, Table 3 shows the number of times each BCT was used in both effective and ineffective studies.

This comparison revealed that, among the top five most used BCTs in the studies, feedback and monitoring (60%-40%) along with associations (57%-43%) have been used homogeneously in effective and ineffective studies, goals and planning (79%-21%) and personalization (71%-29%) have improved the effectiveness of the interventions since they have a higher presence in effective than in ineffective studies, and shaping knowledge (42%-58%) had a lower presence in effective than ineffective studies.

It was observed that 2 of the main BCTs (goals and planning, and associations) used on effective interventions were present in 100% of the adherence-oriented studies for mobile apps and in none of the ineffective studies [30,40,42,52]. Meanwhile, shaping knowledge was present in 100% of the ineffective studies for medication adherence and in none of the effective ones [31,33,35,37,44,47]. This is a good starting point for the design of a general BCT strategy.

**Table 3:** Behavior change techniques used in effective and ineffective studies (N=24).

Behavior change technique	Effective studies (≥80% adherence rate), n (%)	Ineffective studies (<80% adherence rate), n (%)
Feedback and monitoring	12 (50)	8 (33)
Goals and planning	11 (46)	3 (13)
Associations	8 (33)	6 (25)
Shaping knowledge	5 (21)	7 (29)
Personalization	5 (21)	2 (8)
Regulation	4 (17)	3 (13)
Reward and threat	4 (17)	1 (4)
Social support	4 (17)	1 (4)
Comparison of behavior	2 (8)	3 (13)
Natural consequences	3 (13)	0 (0)
Repetition and substitution	2 (8)	0 (0)
Antecedents	1 (4)	1 (4)
Comparison of outcomes	0 (0)	1 (4)

## Discussion

### Principal Findings

We answered three RQs (defined in the Methods section) related to BCTs used in mHealth interventions. To answer RQ1, the taxonomy of BCTs, proposed by Michie et al [24] and which has been updated over the years [27], was identified. Based on this, the BCTs used in the selected studies were extracted and coded to the 18 categories to improve the understanding and meaning of the comparisons. We found that the most frequently used BCTs in mHealth interventions were *feedback and monitoring*, *goals and planning*, *associations*, *shaping knowledge*, and *personalization*. Similar BCTs were also found to be common in reviews of mHealth interventions targeting physical activity and sedentary behaviors, lifestyle, and medication adherence for CCM [59,60].

For RQ2, the categories of BCTs with the highest presence in effective studies were goals and planning, associations, feedback and monitoring, and personalization (as described in the Results section). However, in line with other reviews that found mixed effectiveness in mHealth outcomes [15,27], they suggested that the need remains to improve the use of these and many common BCTs for better outcomes. Possible reasons for the mixed effects of BCTs include the need for details on the specific BCTs that are used in the studies since current mHealth interventions often lump together a multitude of BCTs, making it difficult to discern the characteristics that lead to a study being effective or not and for more specific mHealth intervention content for different population subgroups (eg, those with specific mental health disorders such as anxiety) who may react differently compared to those with healthy mental health in an intervention setting, such as patients with coronary heart disease. This was reflected in the finding that some BCTs did not have the same effects for all groups, with those with higher levels of depression or anxiety deriving less benefit from some BCTs.

Regarding RQ3, considering the responses for RQ1 and RQ2, it was observed that, although some studies in the mHealth context have evaluated user adherence as a complementary outcome resulting from their implementation of BCTs, none of the reviewed studies applied BCTs for adherence to data reporting or use of the mHealth app; the studies generally aimed to improve outcomes for activity in chronically ill or healthy people. However, designing an approach that combines the BCTs most effective for adherence (feedback and monitoring, goals and planning, associations, and personalization), previously identified in RQ2, could be helpful to improve data reporting in mHealth apps.

### Limitations

Several limitations were found that are important to highlight. This review focused on mHealth interventions that include BCTs and how they affect user adherence. As a result, we found a lack of heterogeneity in the sample of results, evidencing that most of these interventions do not focus on improving user adherence to data reporting or to the app but rather on achieving an improvement in the objective of the study, whether it is an improvement in lifestyle behaviors or CCM.

In addition, designing mHealth apps is relatively new, and there is little agreement regarding best practices. Researchers are still trying to understand how and why BCTs lead to positive health outcomes when delivered through apps. There is growing evidence to support the effectiveness of mHealth interventions on health outcomes. Even though the evidence is growing, it is still relatively weak. This may be in part due to the difficulty of designing and conducting rigorous studies on mHealth interventions, even more in the context of the COVID-19 pandemic.

Additionally, the BCT taxonomy approach used to summarize the characteristics of interventions still has some limitations, despite the inclusion of the extension proposed by Dugas et al [27]. This taxonomy allows for the coding of different BCTs but does not assess the intensity or dosage of interventions. Nevertheless, the taxonomy works perfectly well as an excellent starting point and a standard to systematically describe an mHealth intervention.

Finally, we found that most studies are small single-arm studies with short follow-up periods. Future research should focus on studies with longer follow-up periods to determine the effectiveness of mHealth interventions on behavior change.

### Conclusions

mHealth interventions to improve lifestyle behaviors and CCM have become popular in recent years, improving along with different technologies. Although SMS text message–based mHealth interventions remained popular for their proven high results in terms of effectiveness, this review suggests that mHealth is progressively moving toward the implementation of mobile apps and wearable interventions, replacing SMS text messaging with notifications. This review also revealed that mHealth is being applied to address a diversity of lifestyle behaviors and health outcomes, showing its applicability to a variety of health care contexts, with one of its focuses being medication adherence. On the other hand, we found that the most frequently used BCTs in mHealth interventions were feedback and monitoring, goals and planning, associations, shaping knowledge, and personalization. However, this does not necessarily imply that they are the most effective, so we conducted further analysis that found that frequently used BCTs in ineffective studies are often well supported in the health behavior change literature [61,62], suggesting a need to better understand best practices for applying such techniques and to obtain details on the specific BCTs used in mHealth interventions.

Congruent with other reviews that found heterogeneous effectiveness of mHealth, the results also suggested that there remains a need to optimize the use of BCTs or find a better combination for better outcomes, it is intended that this review could help identify the most appropriate techniques to improve adherence to data reporting and thus design an optimal strategy while taking into account the differences among population subgroups, pointing to the need to go beyond the idea that “one-size-fits-all.” As a small step forward, more sophisticated technologies such as wearable activity trackers and wireless sensors have been included in mHealth interventions.

## Appendix

Multimedia Appendix 1 Mobile health studies describing the sample criteria (population), the sample size (N), the study duration (duration), the technology used (tech), and results.

Multimedia Appendix 2 Behavior change techniques in mobile health studies.

## Abbreviations

BCT: behavior change technique
CCM: chronic condition management
mHealth: mobile health
PRISMA: Preferred Reporting Items for Systematic Reviews and Meta-Analyses
RQ: research question
WHO: World Health Organization
