# Supplementary material for: mHealth Apps Using Behavior Change Techniques to Self-report Data: Systematic Review
Source: JMIR Mhealth Uhealth. 2022 Sep 9;10(9):e33247. doi: 10.2196/33247 (PMC9508675; doi:10.2196/33247)
Supplement: Multimedia Appendix 1 [file mhealth_v10i9e33247_app1.doc]

Table S1. mHealth studies describing the sample criteria (Population), the sample size (N), the study duration (Duration), the technology used (Tech), and results.

| Authors | Population | N | Dura-tion^a^ | Study description | Tech | Results | Outcomes |
| --- | --- | --- | --- | --- | --- | --- | --- |
|  |  |  |  |  |  |  |  |
| Klasnja P, et al. (2021) [30] | Bariatric surgery patients | 51 | 4 | Pilot trial (BariFit) to help bariatric patients increase PA^b^ in order to maximise their weight loss and maintenance | Wearable activity tracker (Fitbit) and custom SMS^c^ | Adherence to Fitbit wear, weightings, diet logging was over 95%. Participants increased PA by 1,866 steps from baseline to end-of-study | 1. Adherence to using study equipment.  2. PA increase |
| Jahan Y, et al. (2020) [31] | Individuals with  HTN ^d^ | 420 | 5 | Parallel-group trial to evaluate self-reported behaviour changes (salt intake, fruits and vegetables intake, PA, BP ^e^ and body weight monitoring) | SMS and in-person health education | The adherence rates were significantly higher among the control group participants (39.8%) in comparison to the intervention group participants (36.4%) | 1. Evaluation of self-reported behaviour changes (salt, fruits and vegetables intake, PA, BP, and body weight).  2. measurements of actual intake, blood glucose level, BP values, and quality of life |
| Eaton C, et al. (2020) [32] | Young adults with chronic kidney disease diagnosis, current  anti-hypertensive medication prescription  (pill form only) | 34 | 2 | Pilot trial to monitor adherence through electronic pill bottles and study text messages sent to the patients | SMS | Overall mean adherence was 76.76% in the Reminder-only Message active control group and 76.00% in the Reminder+COM-B^f^ Message intervention group | 1. Daily electronically monitored medication adherence.  2. Pre-post participant surveys of adherence |
| Mata J, et al. (2020) [33] | Adult patients with colonic or rectal diseases planned for surgical resection | 97 | 3 | Parallel-two-group trial divided into: (1) standard preoperative education and sham intervention, (2) standard preoperative education and the use of a mobile device app for postoperative education and self-assessment of recovery | Mobile app on iPad | Mean overall adherence was similar between groups: 59% in the intervention group and 62% in the control group | 1. Mean adherence (%): mobilization, gastrointestinal motility stimulation, breathing exercises, consumption of oral liquids and nutritional drinks |
| Mary R Janevic, et al. (2020) [34] | African American adults with chronic musculoskeletal pain | 51 | 2 | Pilot trial to evaluate the acceptability of a commercially available tracker and three alternative modes of reporting daily steps. Study participants wore trackers, alternately reporting daily step counts via text messages, automated telephone calls, and syncing (two weeks each) | Wearable activity tracker and mHealth reporting (SMS, Interactive voice response and Fitbit mobile app) | Text reporting yielded 79% adherence vs 69% each for automated calls and synchronizing. Intervention participants did not show greater improvement in functioning or walking than controls | 1. Changes in pain interference  2. Physical function  3. Social participation  4. Walking frequency and duration |
| Fico G, et al. (2020) [35] | Type 1 Diabetes and type 2 Diabetes patients | 20 | 1 | Controlled trial to evaluate the use and acceptance of a self-management system (METABO) for diabetes developed with User Centered Design Principles in the community settings | Mobile app and a Desktop-based app | The adherence to PA and food prescriptions (in the latter case concerning the number of meals entered) improved slightly. The intake of calories did not change throughout the trial. Mean adherence was 65% | 1. Blood glucose levels  2. Medication and food intake habits  3. PA |
| Chandler, et al. (2019) [36] | Hispanic adults with hypertension | 54 | 9 | Two-arm efficacy trial addressing medication adherence and BP control with uncontrolled HTN and poor medication adherence, including an experimental (SMASH) group and an enhanced standard care group | Mobile app (Android-iOS) and an electronic medication tray | Average medical regimen adherence, as indicated by time-stamped medication intake and BP monitoring for the SMASH group, ranging from 89.1 to 95.2% | 1. Medication adherence  2. BP control |
| Coorey, et al. (2019) [37] | Patients with moderate-high risk of cardiovascular disease, and their general practitioners | 397 | 12 | Controlled trial to explore effectiveness of persuasive software design for healthier behaviour | Web app integrated with primary health care electronic record | Improved medication adherence (31.8%); improved mental health and well-being (40%); higher PA (47%); and healthier eating (61%). Users of the interactive features reported benefiting from personalised cardiovascular disease risk score (73%); goal tracking (69%); risk factor self-monitoring (52%) and receipt of motivational health tips (54%) | 1. Medication adherence  2. Mental health and well-being  3. PA and healthier eating |
| Hovland Tanneryd, et al. (2019) [38] | Patients diagnosed with heart failure | 72 | 6 | Controlled trial to evaluate a home-based tool for heart failure that provides education, symptom monitoring and titration of diuretics | Specialised tablet connected wirelessly to a weight scale | Improvement in self-care by 27% and the median system adherence was 94% | 1. Heart failure related in-hospital days  2. Self-care behaviour  3. System adherence |
| Morawski K, et al. (2018) [39] | Adults with a systolic BP of 140 mmHg or greater receiving treatment with first-line antihypertensive medications | 411 | 3 | Two-arm clinical trial to determine if the mobile app (MEDISAFE) improves self-reported medication adherence and BP control | Mobile app | Parkinson’s tracker app significantly improved adherence to 79% (MMAS-8 6.3), compared to 72% (MMAS-8 5.74) of the treatment as usual, without confounding effects of gender, number of comorbidities and age | 1. Self-reported medication adherence  2. Change in systolic BP |
| Svendsen M.T, et al. (2018) [40] | Patients with mild-to-moderate psoriasis | 134 | 1 | Controlled trial to evaluate whether a study-specific app improves adherence and reduces psoriasis symptoms compared with standard treatment | Mobile app and an electronic monitor chip synchronised with the app via Bluetooth in the foam dispensers | More patients in the intervention group were adherent to Cal/BD cutaneous foam than those in the non-intervention group at week 4 (65% vs. 38%) | 1. Adherence to study-specific app  2. Psoriasis severity  3. Quality of life |
| Labovitz D, et al. (2017) [41] | Adults with recently diagnosed ischemic stroke receiving any anticoagulation | 28 | 3 | Parallel-group study to evaluate the use of an artificial intelligence platform on mobile devices in measuring and increasing medication adherence in stroke patients on anticoagulation therapy | Mobile app | Mean cumulative adherence based on the artificial intelligence platform was 90.5%. Plasma drug concentration levels indicated that adherence was 100% (15 of 15) and 50% (6 of 12) in the intervention and control groups, respectively | 1. Measuring and increasing medication adherence in stroke patients on anticoagulation therapy |
| Lakshminaray R, et al. (2017) [42] | Parkinson’s disease patients | 158 | 4 | Multi-centre controlled trial to assess the impact of using a smartphone-based Parkinson’s tracker app to promote patient self-management, enhance treatment adherence and quality of clinical consultation | Mobile app | Parkinson’s tracker app significantly improved adherence to 79% (MMAS-8 6.3), compared to 72% (MMAS-8 5.74) of the treatment as usual, without confounding effects of gender, number of comorbidities and age | 1. Self-reported score of adherence to treatment  2. Quality of life |
| Mertens A, et al. (2016) [43] | Elderly patients with coronary heart disease | 24 | 8 | Crossover-design study to analyse whether a mobile application on a tablet aimed at supporting drug intake and vital sign parameter documentation affects adherence | Mobile app on iPad | The mean for subjectively assessed adherence before the study was 89%. After both interventions, there was an increase, higher after the intervention phase (96%) than after the comparative phase (94%) | 1. Drug intake  2. Vital sign parameter documentation |
| Recio-Rodriguez J, et al. (2016) [44] | Sedentary and active adults without heart diseases | 833 | 3 | Multicenter controlled trial to evaluate the short-term effects of adding an app in support of standardised counselling to increase PA and adherence to the Mediterranean diet | Mobile app | Adherence rate to the Mediterranean diet increased in both groups: from 34.2% to 42.6% in app+counseling and from 28.5% to 38.9% in counselling only group | 1. PA  2. Adherence to the Mediterranean diet |
| Pfaeffli Dale L, et al. (2015) [45] | Adults diagnosed with coronary heart disease | 123 | 6 | Two-arm controlled trial to investigate the effectiveness of a mHealth-delivered comprehensive cardiac rehabilitation to improve adherence to recommended lifestyle behaviours. The control group were encouraged to attend centre-based cardiac rehabilitation and the intervention group received a personalised mHealth program | Fully automated daily SMS and a supporting web app | Fully automated daily SMS and a supporting web app | 1. Adherence to healthy lifestyle behaviours (self- reported composite)  2. Medication adherence score, self-efficacy, illness perceptions, and anxiety and/or depression |
| Ammenwert, et al. (2015) [46] | Hospitalised patients for acute myocardial infarction and/or percutaneous coronary intervention | 25 | 4,5 | Formative evaluation with two telemonitoring phases and one interim phase (MyCor). Patients measured BP and weight daily, use of pedometer for continuous foot-step counting, documented drug intake and subjective well-being on the smartphone once daily; received tailored goal setting, education, feedback, and regular clinic visits | Mobile app, BP meter, pedometer and a supporting web interface | Adherence to daily measurements of 86% and 77% in the two tele monitoring phases. Adherence to medication between 80% and 87%. Pre-defined goals for PA were reached between 73% and 86% of days, respectively | 1. Medication management  2. Lifestyle changes |
| Naimark J, et al. (2015) [47] | Adults from the south and center of Israel | 99 | 2,75 | Controlled trial to compare people receiving a new app (eBalance) with people who got an introductory lecture on healthy lifestyle alone, weight change, nutritional knowledge, and PA, and to identify predictors of success for maintaining a healthy lifestyle | Mobile app | Adherence rate was 56%, calculated for all app users including light and heavy users. Success score (i.e., represents the success in maintaining a healthy lifestyle) was higher among the app group (68%) compared with 36% in the control group | 1. Physical activity  2. Weight  3. Nutritional knowledge  4. Diet quality |
| Hammonds T, et al. (2015) [48] | College students who had a current prescription for an antidepressant | 57 | 1 | Parallel-group clinical trial to determine if medication reminding via smartphone app increases adherence to antidepressant medications in college students | Mobile app | The adherence rate was 76% for the treatment group and 70.4% for the control group | 1. Medication adherence |
| Goldstein, et al. (2014) [49] | Older adults with heart failure | 58 | 1 | Controlled trial to evaluate a telehealth intervention (an electronic pillbox) and a mHealth intervention (an app on a smartphone) for improving medication adherence. Each intervention was divided into two groups, with and without reminders | Mobile app, SMS and electronic pillbox | Overall adherence rate was 78%. Reminding did not improve adherence. Patients preferred the m-health approach | 1. Medication adherence |
| Santo, et al. (2018) [59] | Patients with Coronary Heart Disease | 166 | 3 | Randomised clinical trial to evaluate the effectiveness and feasibility of using medication reminder applications (apps) to improve medication adherence compared with usual care in patients with coronary heart disease. An additional aim was to examine whether an app with additional features improved adherence further | Mobile app | Patients using an app had higher adherence (88.9%) compared with the usual care group (82.9%) | 1. Medication adherence  2. BP and cholesterol levels |
| Varnfield, et al. (2014) [60] | Post-myocardial infarction patients | 120 | 7 | Randomised controlled trial to investigate the effect of a smartphone-based home service delivery of cardiac rehabilitation and health outcomes compared with a traditional, centre based programme | Mobile app | Mobile app for cardiac rehabilitation had significantly higher uptake (80% vs 62%), adherence (94% vs 68%) and completion (80% vs 47%) rates than traditional cardiac rehabilitation | 1. Uptake and adherence  2. Changes in clinical outcomes (modifiable lifestyle factors, biomedical risk factors and health-related quality of life) |
| Hartman, et al. (2018) [61] | Female breast cancer survivors | 42 | 3 | Randomized controlled trial to examine patterns of Fitbit use and activity and their relationships with success in the intervention based on ActiGraph-measured moderate to vigorous PA | Wearable activity tracker, mobile app | Adherence to wearing the Fitbit was high and stable, with a mean of 88.13%. Greater adherence to wearing the Fitbit was associated with greater increases in ActiGraph-measured | 1. PA  2. Tracker and app adherence |
| Párraga-Martínez, et al. (2017) [62] | Diagnosed with hypercholesterolemia whether receiving prior therapy or not | 358 | 12 | Randomized parallel-group clinical trial. Intervention patients received written information on disease and its treatment and self-completed registration cards on adherence. Text messages with summaries of recommendations, reminders of appointments, and in-person consultations | SMS | Adherence at the end of the study: 78.5% intervention, 64.9% usual care | 1. Cholesterol levels  2. Adherence to recommendations on lifestyle and drug treatment  3. Beliefs and expectations about preventive recommendations |

^a^Duration in months.

^b^PA: physical activity.

^c^SMS: short message service.

^d^HTN: hypertension.

^e^BP: blood pressure.

^f^COM-B: ’capability’, ’opportunity’, ’motivation’ and ’behaviour’ model.

^g^MMAS-8: Morisky Medication Adherence Scale.
