# Supplementary material for: mHealth Apps Using Behavior Change Techniques to Self-report Data: Systematic Review
Source: JMIR Mhealth Uhealth. 2022 Sep 9;10(9):e33247. doi: 10.2196/33247 (PMC9508675; doi:10.2196/33247)
Supplement: Multimedia Appendix 2 [file mhealth_v10i9e33247_app2.doc]

Table S2. BCTs in mHealth studies.

| Authors | Goals and planning | Feedback and monitoring | Social support | Shaping knowledge | Natural consequences | Comparison of behaviour | Associations | Repetition and substitution | Comparison of outcomes | Reward and threat | Regulation | Antecedents | Personalisation | **Total by study** |
| --- | --- | --- | --- | --- | --- | --- | --- | --- | --- | --- | --- | --- | --- | --- |
|  |  |  |  |  |  |  |  |  |  |  |  |  |  |  |
| Klasnja P, et al. (2021) [30] | ✓ | - | - | - | - | - | ✓ | - | - | - | ✓ | - | ✓ | 4 |
| Jahan Y, et al. (2020) [31] | ✓ | ✓ | - | ✓ | - | - | ✓ | - | - | - | - | - | - | 4 |
| Eaton C, et al. (2020) [32] | - | - | - | ✓ | ✓ | - | - | - | - | - | - | - | - | 2 |
| Mata J, et al. (2020) [33] | - | ✓ | - | ✓ | - | - | - | - | - | - | - | - | - | 2 |
| Mary R Janevic, et al. (2020) [34] | - | ✓ | - | - | - | - | - | - | - | ✓ | - | - | - | 2 |
| Fico G, et al. (2020) [35] | - | ✓ | - | ✓ | - | ✓ | ✓ | - | ✓ | - | ✓ | ✓ | - | 7 |
| Chandler, et al. (2019) [36] | ✓ | ✓ | ✓ | ✓ | - | ✓ | ✓ | - | - | ✓ | - | - | - | 7 |
| Coorey, et al. (2019) [37] | - | ✓ | ✓ | ✓ | - | ✓ | ✓ | - | - | ✓ | ✓ | - | - | 7 |
| Hovland Tanneryd, et al. (2019) [38] | - | ✓ | - | ✓ | - | - | ✓ | - | - | - | ✓ | - | ✓ | 5 |
| Morawski K, et al. (2018) [39] | ✓ | ✓ | ✓ | - | - | - | - | ✓ | - | - | - | - | ✓ | 5 |
| Svendsen M.T, et al. (2018) [40] | - | ✓ | - | - | - | - | - | - | - | - | - | - | ✓ | 2 |
| Labovitz D, et al. (2017) [41] | ✓ | ✓ | - | - | - | - | ✓ | - | - | - | ✓ | - | - | 4 |
| Lakshminaray R, et al. (2017) [42] | ✓ | ✓ | - | - | - | - | ✓ | - | - | - | - | - | ✓ | 4 |
| Mertens A, et al. (2016) [43] | ✓ | ✓ | - | - | - | - | - | - | - | - | - | - | - | 2 |
| Recio-Rodriguez J, et al. (2016) [44] | ✓ | ✓ | - | ✓ | - | - | ✓ | - | - | - | - | - | - | 4 |
| Pfaeffli Dale L, et al. (2015) [45] | - | ✓ | - | ✓ | - | - | ✓ | - | - | - | - | - | ✓ | 4 |
| Ammenwert, et al. (2015) [46] | ✓ | ✓ | - | ✓ | - | - | - | - | - | - | - | - | - | 3 |
| Naimark J, et al. (2015) [47] | ✓ | ✓ | - | ✓ | - | ✓ | ✓ | - | - | - | ✓ | - | - | 6 |
| Hammonds T, et al. (2015) [48] | ✓ | - | - | - | - | - | ✓ | - | - | ✓ | - | - | ✓ | 4 |
| Goldstein, et al. (2014) [49] | ✓ | ✓ | - | ✓ | - | - | - | - | - | - | - | - | - | 3 |
| Santo, et al. (2018) [59] | - | ✓ | ✓ | - | - | - | ✓ | - | - | - | - | - | - | 3 |
| Varnfield, et al. (2014) [60] | ✓ | ✓ | - | - | ✓ | - | - | - | - | - | ✓ | - | - | 4 |
| Hartman, et al. (2018) [61] | ✓ | ✓ | ✓ | - | ✓ | ✓ | - | ✓ | - | ✓ | - | ✓ | - | 8 |
| Párraga-Martínez, et al. (2017) [62] | - | - | - | - | - | - | ✓ | - | - | - | - | - | - | 1 |
| **Total by category** | 14 | 20 | 5 | 12 | 3 | 5 | 14 | 2 | 1 | 5 | 7 | 2 | 7 |  |
